# Modulating the Gut Microbiome in Multiple Sclerosis Management: A Systematic Review of Current Interventions

**DOI:** 10.3390/jcm12247610

**Published:** 2023-12-11

**Authors:** Anthi Tsogka, Dimitrios K. Kitsos, Konstantina Stavrogianni, Vasileios Giannopapas, Athanasios Chasiotis, Niki Christouli, Georgios Tsivgoulis, John S. Tzartos, Sotirios Giannopoulos

**Affiliations:** 1 Second Department of Neurology, Attikon University Hospital, School of Medicine, National and Kapodistrian University of Athens, 157 72 Athens, Greece; tsogka.anthi@gmail.com (A.T.); dkitsos@icloud.com (D.K.K.); stavrogianni.k@gmail.com (K.S.); bgiannopapas@gmail.com (V.G.); thanosch1@gmail.com (A.C.); nichristouli@gmail.com (N.C.); tsivgoulisgiorg@yahoo.gr (G.T.); jtzartos@gmail.com (J.S.T.); 2 Department of Physiology, Faculty of Medicine, School of Health Sciences, University of Ioannina, 451 10 Ioannina, Greece; 3 Department of Physical Therapy, University of West Attica, 122 43 Attica, Greece; 4 Laboratory of Neuromuscular and Cardiovascular Study of Motion-LANECASM, University of West Attica, 122 43 Attica, Greece

**Keywords:** gut microbiome, multiple sclerosis, probiotics, dietary protocol, fecal microbiota transplantation, microbiota

## Abstract

This review attempted to explore all recent clinical studies that have investigated the clinical and autoimmune impact of gut microbiota interventions in multiple sclerosis (MS), including dietary protocols, probiotics, fecal microbiota transplantation (FMT), and intermittent fasting (IF). **Methods:** Thirteen studies were held between 2011 and 2023 this demonstrated interventions in gut microbiome among patients with MS and their impact the clinical parameters of the disease. These included specialized dietary interventions, the supply of probiotic mixtures, FMT, and IF. **Results:** Dietary interventions positively affected various aspects of MS, including relapse rates, EDSS disability scores, MS-related fatigue, and metabolic features. Probiotic mixtures showed promising results on MS-related fatigue, EDSS parameters, inflammation; meanwhile, FMT—though a limited number of studies was included—indicated some clinical improvement in similar variables. IF showed reductions in EDSS scores and significant improvement in patients’ emotional statuses. **Conclusions:** In dietary protocols, clinical MS parameters, including relapse rate, EDSS, MFIS, FSS, and MSQoL54 scales, were significantly improved through the application of a specific diet each time. Probiotic nutritional mixtures promote a shift in inflammation towards an anti-inflammatory cytokine profile in patients with MS. The administration of such mixtures affected disability, mood levels, and quality of life among patients with MS. FMT protocols possibly demonstrate a therapeutic effect in some case reports. IF protocols were found to ameliorate EDSS and FAMS scores. All interventional means of gut microbiome modulation provided significant conclusions on several clinical aspects of MS and highlight the complexity in the relationship between MS and the gut microbiome.

## 1. Introduction

Multiple sclerosis (MS), an autoimmune disease of the central nervous system (CNS), is characterized by demyelination, axonal damage, and progressive neurologic disability [1]. While genetic factors (e.g., certain human leucocyte antigen (HLA) class II haplotypes) have emerged as strong candidates associated with the disease, the exact nature of contributing environmental factors, particularly those related to the gut microbiota, remains an area of growing interest [2].

Gut-associated lymphoid tissue (GALT) is a vital component of the gastrointestinal system, serving a dual role in protecting against harmful microbes while also regulating the gut’s normal flora. Within the GALT, immune cells help maintain the intestinal epithelium’s integrity by facilitating antigen transport, presentation, and activation of T and B cells [3,4,5]. Simultaneously, the gut’s physiology is influenced by the autonomous nerve system (ANS) and the hypothalamus–pituitary axis (HPA), impacting motility, secretion, and gut microbiota. Additionally, the central nervous system (CNS) plays essential roles in neurotransmitter secretion within the gastrointestinal tract [6,7]. These intricate interplays underscore the significant impact of these interactions on overall health.

**Figure 1:**
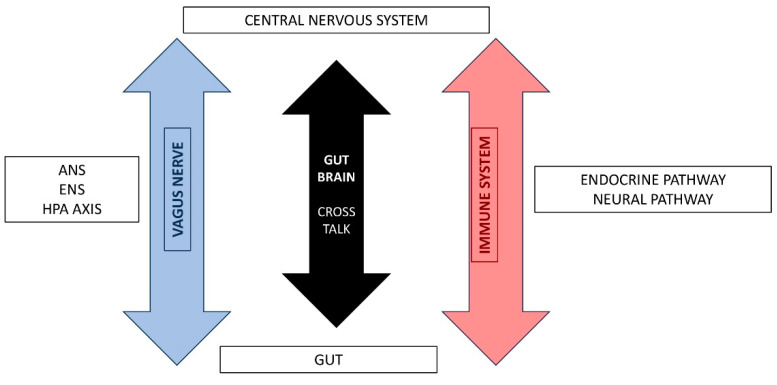
Gut-Brain Axis pathways.

Over the past decade, a large number of animal and preclinical studies have proven that gut microbiota is involved in triggering bidirectional signaling through the gut–brain axis. This term describes the physiology concept that includes all afferent and efferent molecular pathways, endocrine, immune, neural, and metabolic, that participate in signaling between the GI and the CNS [6,7] (Figure 1).

The gut microbiome plays an indispensable role in the development of immune function, a prerequisite for the emergence of autoimmune diseases. However, the precise nature of this role in initiating and advancing the MS remains unclear. One area of particular interest lies in discerning the disparities between the microbiota of healthy individuals and those affected by MS. Such differences hold substantial promise for shedding light on the pathophysiology of MS [8].

Recent findings from animal studies have unveiled a compelling connection between the gut microbiota’s composition and its influence on the pathogenesis and progression of MS [9,10]. MS may affect the balance between pro-inflammatory and anti-inflammatory cytokines, may stimulate the production of lymphocytes or activate autoimmunity via the molecular mimicry phenomenon [3,6]. Given the microbiota’s demonstrated modulatory role in autoimmune diseases, it emerges as an exceptionally promising therapeutic target for the management of MS and the inhibition of disease progression [11].

### Gut-Microbiome-Based Therapeutic Interventions in MS

As mentioned earlier, it is possible that the gut microbiota act as modulators in autoimmune diseases. Consequently, they could be considered as a potential therapeutic target, in patients with MS, in order to inhibit disease progression and manage symptoms. In some cases, therapeutic interventions may cause side effects or prove to be ineffective, while in other cases, beneficial results have been demonstrated.

Diet, a critical factor in shaping gut microbiota, plays a substantial role in MS. Western dietary patterns, characterized by high saturated fat and carbohydrate intake, can induce structural changes in the gut flora, thereby promoting chronic inflammation through alterations in microbiome gene expression. The adoption of diets rich in salt, animal fats, and carbohydrates, coupled with sedentary lifestyles, has been associated with the exacerbation of MS symptoms and the promotion of dysbiosis in the intestinal microflora [3,6,9].

Studies investigating the role of the intestinal barrier have yielded compelling evidence that underscores the significant protective potential of probiotics in MS [12]. Probiotics are microorganisms that are renowned for their health benefits that serve multifaceted functions, including the preservation of the intestinal barrier, the production of antimicrobial peptides, and the reinforcement of the host’s immune system. Certain microorganisms, such as *Lactobacillus* and *Prevotella species*, have exhibited potential in reducing symptom severity by mediating the production of anti-inflammatory interleukin-10 (IL-10) and supporting regulatory T cells (Treg); however, others, like *Porphyromonas gingivalis*, have been linked to exacerbated EAE severity through the promotion of pro-inflammatory processes and gliosis [3,6]. Furthermore, Omega-3 polyunsaturated fatty acids (PUFAs) are antioxidants linked to ameliorating neurodegeneration in MS and seem to play a vital role in disease pathophysiology [13]. PUFA-rich diets have been associated with a low incidence of MS, a reduction in the frequency of relapses but no effect on the progression of MS [14]. Matrix metallopeptidase-9 (MMP-9) is a specific marker associated with inflammation and neurodegeneration in patients with MS. Among PUFAs, alpha-linolenic acid (ALA) is linked to a reduced incidence of MS [15]. A recent randomized controlled trial conducted on 51 patients with RRMS demonstrated that a unique dietary formula (Neuroaspis^®^PLP10), a combination of specific bioactive molecules, the omega-3 PUFAs DHA and EPA, the omega-6 PUFAs linoleic acid and gamma-linolenic acid, and several vitamins such as vitamin E and tocopherol, may act protectively against functional deterioration of patients with RRMS [16].

Fecal microbiota transplantation (FMT), a pioneering approach that aims to restore gut microbiome function by replacing it with a healthy counterpart, has demonstrated remarkable effectiveness in conditions such as Clostridium difficile infection and inflammatory bowel disease (IBD). Its potential application in MS remains relatively unexplored, with limited clinical trials involving small patient cohorts. While case reports suggest improvements in walking ability post-FMT, adverse effects have also been noted [3,6].

Intermittent fasting (IF), characterized by dietary protocols involving time-restricted and quantity-regulated food intake, has shown promise in reducing pro-inflammatory cytokines in vitro [10]. Clinical studies have yielded inconsistent results regarding its benefits for patients with MS and the data available on the influence of gut microbiota in MS are notably limited [6,10].

This systematic review aims to comprehensively explore gut-microbiome-based interventional studies in patients with MS (pwMS). By examining diverse approaches such as dietary modifications, probiotic supplements, FMT, and IF, this review seeks to shed light on the potential impact of these interventions on the course and manifestation of MS.

## 2. Methods

**Figure 2:**
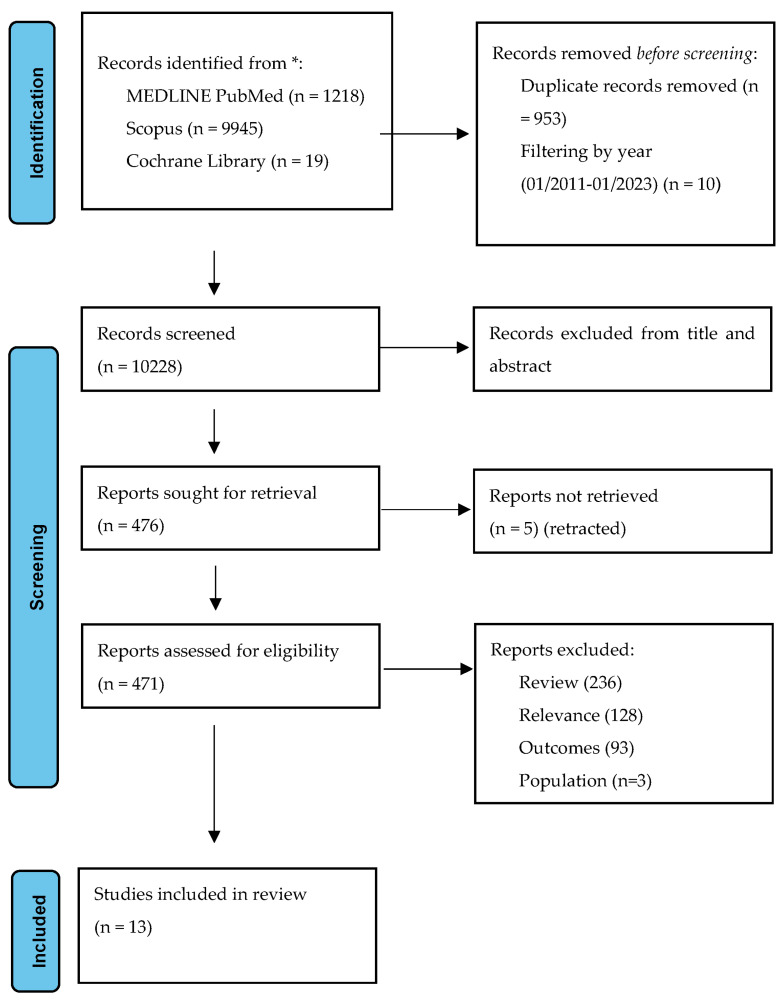
Prisma flowchart: study selection algorithm based on the inclusion–exclusion criteria.

The systematic review is reported according to the Preferred Reporting Item for System and Meta-Analysis (PRISMA) guidelines [17]. A systematic literature review was conducted to identify eligible records based on pre-defined PICO criteria (population—adult patients with MS; intervention—dietary modification, probiotics, fecal microbial transplantation; comparison—patients with MS, healthy controls; observation—EDSS, biomarkers) by two independent reviewers (A.T. and D.K.K.). We searched the MEDLINE PubMed, Scopus, and Cochrane Library using a predefined search algorithm. The library database was accessed covering the period from 1 January 2011 to 1 June 2023. The inclusion criteria consisted of the following: (a) patients with confirmed multiple sclerosis; (b) participants’ age > 18 years; (c) gut microbiome intervention; (d) clinical outcome; (e) study design—randomized control trials, case–control studies, observational studies, case series, and case reports. Any disagreements were resolved by the senior author (S.G.). The systematic review is registered in the Open Science Framework (https://osf.io/p57ae (accessed on 9 November 2023)). The results of the systematic review are presented in Figure 2. Eligible studies underwent quality assessment with the use of the ROBINS-I tool for non-randomized control trials [18]. The results are presented in the form of a traffic light plot.

## 3. Results

### 3.1. Systematic Analysis 

**Figure 3:**
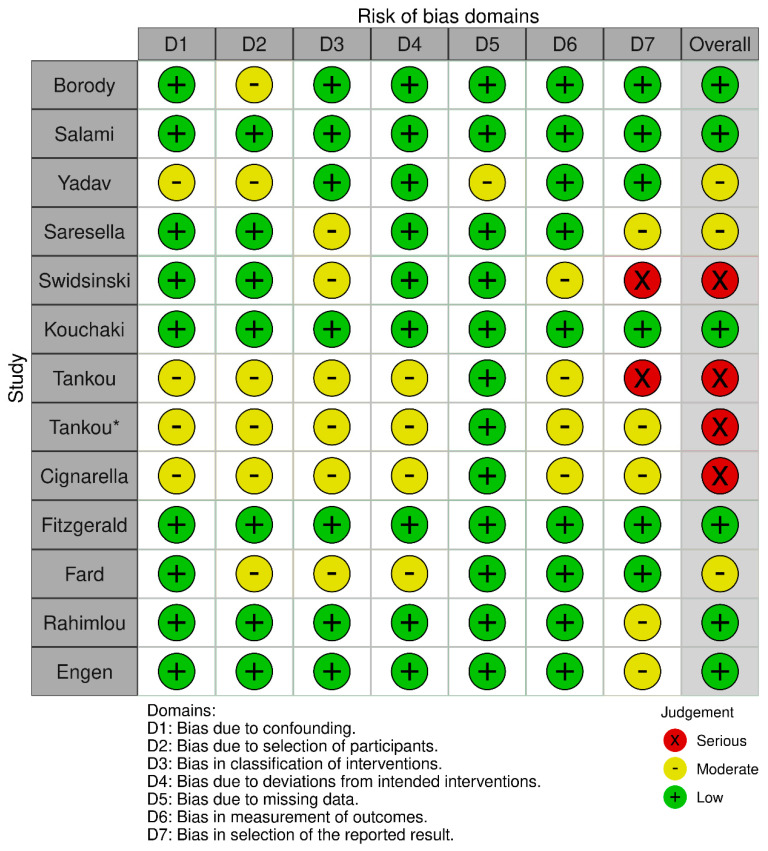
Robins-I risk of bias traffic plot. There are two studies by Tankou et al. [25,26] so in order to create the traffic plot the earliest study was marked by an asterisk.

A total of thirteen studies [19,20,21,22,23,24,25,26,27,28,29,30,31] with a moderate–serious risk of bias were included in the systematic review (Table 1, Figure 3). Four studies examined the effectiveness of diet modification, two assessed the effectiveness of intermittent fasting, five assessed the effectiveness of probiotic supplementation, and two assessed the effectiveness of fecal microbiota transplantation (Table 2).

### 3.2. Dietary Modifications

*Fard and colleagues* [19] aimed to assess the impact of a modified anti-inflammatory diet on fatigue, QoL, and inflammatory markers in patients with RRMS in a randomized clinical trial. A total of 100 patients were divided into either the diet group (receiving an anti-inflammatory diet) or the control group (receiving healthy diet recommendations) for a duration of 12 weeks. The study measured fatigue using the Modified Fatigue Impact Scale (MFIS) and QoL using the Multiple Sclerosis Quality of Life (MSQoL-54). Additionally, anthropometric measures and inflammatory biomarkers, including Interleukin 17 (IL-17), Interleukin 4 (IL-4), and high-sensitivity C-reactive protein (hs-CRP), were assessed. The findings revealed significant improvements in MFIS scores and both the physical and mental components of MSQoL-54 in the diet group compared to the control group (*p < 0.05*). Moreover, the study observed a significant increase in IL-4 levels (*0.05*). No significant changes were observed in IL-17 or hs-CRP levels *(0.05*).

In a pilot study led by *Saresella and colleagues* [20], the researchers investigated the immunological and clinical effects of dietary intervention in two groups of RRMS patients. One group (n = 10) followed a high-vegetable/low-protein diet (HV/LP diet group), while the other group (n = 10) adhered to a “Western Diet” (WD group) for a minimum of 12 months. After analyzing both the composition of gut microbiota and immunological profiles following this intervention, it was observed that the HV/LP diet group had notably higher levels of the *Lachnospiraceae* family, reduced levels of IL-17-producing T CD4+ lymphocytes and T CD4+ lymphocytes expressing PD-1, and an increase in monocytes expressing PD-L1 (*0.05*). In terms of clinical parameters, the HV/LP diet group demonstrated improvements, including a significant reduction in the Expanded Disability Status Scale (EDSS) score and a lower number of disease relapses when compared to the WD group (*0.05*).

In a randomized–controlled study conducted by *Yadav and colleagues* [21], the researchers investigated the effects of adopting a very-low-fat, plant-based diet on radiological, metabolic, and clinical aspects of MS, encompassing disease progression, brain imaging-based disease activity, MS-related symptoms, and overall quality of life (QoL) measures. The study included 61 pwMS, divided into either the diet group (N = 32) or the control group (N = 29). The findings indicated that pwMS in the diet group experienced notable reductions in fatigue levels, as evidenced by lower scores on both the Fatigue Severity Scale (FSS) and the Modified Fatigue Impact Scale (MFIS) (*0.05*). However, when assessing MRI results, the number of relapses, and disability levels measured using the EDSS, there were no statistically significant differences observed between the two groups (*0.05*). Nonetheless, the diet group did exhibit positive improvements at the six-month mark, including enhancements in LDL and total cholesterol levels, as well as insulin levels, and a consistent decrease in BMI (*0.05*).

*Swidsinski and colleague* [22] utilized a case–control study to investigate colonic microbiome synthesis with fluorescence in situ hybridization ribosomal RNA-based FISH probes. The study included 25 RRMS patients and 14 controls. The investigators applied a ketogenic based diet on 10 out of a group of 25 RRMS patients for 6 months and monitored the changes in concentration of 35 bacterial groups at 2, 12, and 23/24 weeks. The results indicate that total concentrations and biodiversity of certain bacterial groups reduced in patients with MS *(0.001*). Specifically, *Roseburia, Bac303 (Bacteroides), and Fprau (Faecalibacterium prausnitzii)* probes were mostly eliminated, while the effects of ketogenic diet demonstrate a fluctuation as in a short-term bacterial concentration were reduced and recovered after 23–24 weeks. The investigators concluded that ketogenic diet normalizes concentration of gut colonic microbiome after a 6-month period. 

### 3.3. Probiotic Nutritional Mixtures

*Rahimlou and colleagues* [23] conducted a study that involved 70 RRMS patients who were randomly assigned to either an intervention group (n = 35) or a control group (n = 35). The study aimed to investigate the effects of multi-strain probiotic supplementation on serum levels of IL-6, BDNF, nerve growth factor (NGF), and aspects of mental health, including depression, fatigue, and pain. The intervention group received two multi-strain probiotic capsules daily for a period of six months, while the control group received a placebo. The results demonstrated that probiotic supplementation led to a statistically significant increase in BDNF levels and a significant decrease in IL-6 levels (*0.001*). The intervention group experienced significant enhancements in mental health parameters, as assessed through various measures, including the General Health Questionnaire-28 (GHQ-28), the Beck Depression Inventory-II (BDI-II), the Fatigue Severity Scale (FSS), and the Pain Rating Index (PRI) (*0.01*). There were no notable differences between the two groups in terms of the EDSS score (*0.05*).

In their 12-week study comprising 60 RRMS patients, who were randomly assigned to either the intervention group (n = 30) or the placebo group (n = 30), *Kouchaki and colleagues* [24] aimed to assess the effects of probiotic supplementation on disability progression, mental health parameters, and metabolic indicators. Probiotic intake led to a noteworthy improvement in EDSS score and a significant reduction in BDI and Depression Anxiety and Stress Scale (DASS) scores, further corroborated by significantly enhanced GHQ-28 scores (*0.01*). Probiotic supplementation also resulted in significant differences in C-reactive protein levels, pointing to reduced inflammation and nitric oxide metabolites levels (*0.01*), reflecting improvements in metabolic health. The study noted differences in malondialdehyde (MDA) levels (*p = 0.04*), indicating favorable changes in oxidative stress. Serum insulin levels significantly decreased, and insulin resistance improved (*0.001*) suggesting enhanced insulin sensitivity and better metabolic health. Total-/HDL-cholesterol levels differed significantly, and HDL-cholesterol levels increased (*p = 0.02*), reflecting favorable lipid profile and enhanced cardiovascular health.

*Tankou and colleagues* [25] showed that the administration of the probiotic nutritious mixture VSL3 was associated with increase in the relative abundance of several species in both controls and pwMS (*p = 0.048*). In addition, VSL3 administration induced an anti-inflammatory peripheral innate immune response (*p = 0.08*), while discontinuation of VSL3 induced a pro-inflammatory immune response characterized by increased frequency of inflammatory monocytes in controls (*p = 0.033*). The authors concluded that the use of probiotic VSL3 can induce changes in the gut microbiota composition that are associated with an anti-inflammatory peripheral innate immune response in controls and pwMS. These immunomodulatory effects did not persist after discontinuation of the VSL3 supplement.

Based on the same protocol, *Tankou and colleagues* [26] showed that another probiotic supplementation lactobacillus–bifidobacterium–streptococcus (LBS) was associated with an enrichment of taxa depleted in MS including genus Lactobacillus in both controls and patients with MS (*0.05*). They also observed a depletion of taxa associated with dysbiosis in MS such as Blautia and Dorea (*0.05*). In addition, the abundance of several Kyoto Encyclopedia of Genes and Genomes (KEGG) pathways was decreased in both controls and patients with MS including pathways related to metabolism, cellular processes, environmental information, and processing and organismal systems (*0.05*). The authors also found changes in stool metabolomics profiles which were associated with decreased expression of MS risk allele HLA.DPB1 (*p = 0.078*) as well as HLA.DPA1 (*p = 0.055*); this was also seen at the immune level where LBS administration induced an anti-inflammatory peripheral immune response characterized by decreased frequency of intermediate monocytes in patients with MS (*p = 0.094*).

In a randomized clinical trial conducted by *Salami and colleagues* [27], a total of 48 RRMS patients were enrolled and randomly assigned to two groups: one receiving probiotics (n = 24) and the other receiving a placebo (n = 24) over a 16-week period. The study aimed to investigate the impact of probiotic intervention on antioxidant levels, metabolic biomarkers, and various mental health parameters. The results demonstrated significant improvements, including reductions in EDSS, BDI, and DASS scores, reflecting diminished disability progression, depressive symptoms, and psychological distress, as well as enhanced overall wellbeing measured by GHQ-28 (*0.05*). Probiotic supplementation was also associated with a notable decrease in malondialdehyde and 8-hydroxy-2′-deoxyguanosine levels, indicative of improved oxidative stress and damage (*0.001*). Additionally, probiotic use led to a significant decrease in IL-6 and high-sensitivity C-reactive protein levels (*0.05*), signifying reduced inflammation, alongside a significant increase in IL-10 levels (*0.001*), suggesting an anti-inflammatory effect.

### 3.4. Fecal Microbiota Transplantation

*Engen and colleagues* [28] presented 1 case report of a RRMS patient who underwent FMT and was followed up for 1 year following the transplantation process. The authors observed increased BDNF serum levels and a significant decrease in scores on the 12-item MS Walking Scale questionnaire (*p* < *0.05*).

*Borody and colleagues* [29] presented three case reports of patients with MS, described diagnostically as “atypical MS”, which received a fixed number of fecal transplantations and were prospectively followed up. None of the three cases presented information regarding the methodology and exact FMT procedure or any data regarding the feces donor. The authors concluded on some general promising results which included “the ability to walk and removal of catheter” in case 1, “the ability to walk and resolution of leg paresthesia” in case 2, and “improvement of constipation and increased energy levels” in case 3. None of the conclusions were based on objective clinical evaluation tools but solely on patient self-assessment.

### 3.5. Intermittent Fasting

*Cignarella and colleagues* [30] conducted a study involving 16 RRMS patients who were experiencing clinical relapse. Out of these, eight participants underwent a 2-week period of intermittent fasting (IF), while the remaining eight followed a controlled diet for the same duration (control group). The study’s findings revealed a significant improvement in both groups in terms of EDSS score, although there was no statistically significant difference observed in Multiple Sclerosis Functional Composite (MSFC) and Symbol Digit Modalities Test (SDMT) scores between the two groups. Additionally, metabolic leptin levels were significantly reduced in the IF group compared to the controls *(0.05*). However, adiponectin and β-hydroxybutyrate levels did not exhibit any statistical difference between the groups (*0.5*). Furthermore, there were no significant differences in gut microbiome bacteria, including *Faecalibacterium, Lachnospiracea incertae sedis*, and *Blautia*, between the two groups *(0.05*).

*Fitzgerald and colleagues* [31] conducted a study involving 36 RRMS patients who were divided equally into three different diet protocols: daily selective restriction (CR) diet, intermittent CR diet, and a weight-stable diet. The study lasted for 8 weeks. The findings revealed that participants randomized to both CR diets experienced improvements in emotional wellbeing and depression components (*0.05*) of the Functional Assessment of MS (FAMS). However, there was a statistically insignificant association observed between the type of diet and the components related to fatigue and QoL as measured by the FAMS. Notably, the daily CR diet was associated with a greater reduction in fat mass compared to the intermittent CR diet (*0.05*), and both CR diets were linked to a decline in cholesterol levels. 

## 4. Discussion

This systematic review aimed to identify and evaluate the different gut microbiome interventions in pwMS. Based on the literature review, the main interventions in MS encompass dietary modifications, IF, probiotic supplementation, and FMT. Our review included 13 studies, with a total of 212 pwMS participants and 200 control subjects. There is increasing evidence from animal studies of a relationship between the type of gut microflora and the pathogenesis and progression of MS [32,33,34,35,36]. The gut microbiota may affect the onset and the progression of MS via affecting the full spectrum of MS pathophysiological mechanisms [9]. MS may affect the balance between pro-inflammatory and anti-inflammatory cytokines, may stimulate the production of lymphocytes, or activate autoimmunity via the molecular mimicry phenomenon [3,13]. Although the role of bacterial pathogens as MS initiators is not entirely clear, it has been established that intestinal microflora can produce various metabolic by-products, known as metabolome, that may enhance autoimmunity [9,37]. 

Dietary modifications, as a therapeutic approach in MS, are rather unexplored. In general, diet affects the composition of the gut microbiota. Western dietary habits consist of saturated fats and carbohydrates that may cause structural changes in gut flora, leading to chronic inflammation, by altering the gene expression of the microbiome [6,9,38,39,40]. A diet rich in salt, saturated fats, and carbohydrates, combined with the absence of any physical exercise, and the presence of stress, prolonged sun exposure, and infection, can lead to increased MS symptoms and sustain a dysbiotic intestinal microflora [40,41]. In all studies that included dietary modifications [19,20,21,22], certain clinical parameters of MS were observed to be significantly affected. More specifically, rr, EDSS, MFIS, FSS, and MSQoL54 scales were significantly improved via applying specific dietary protocols. The results abide with the findings in the aforementioned literature regarding dietary regimes rich in fibers and low in protein and saturated fats. In one study [19], this dietary compound is defined as an “anti-inflammatory diet protocol”. Based on the above, all diet protocols applied may be easily assimilated in real-world daily routines without any real time or financial burden being experienced by the individual. In addition, the application of diet regimes rich in fibers may also have a beneficial effect on certain probable comorbid risk factors for vascular or other incidents [42]. Finally, the adaptation of diet protocols that are rich in fibers may also become an important non-pharmacological ally for patients with MS during the constant struggle with the disability, aside from the pharmacological and rehabilitation applications. 

The most common functions of a probiotic nutritional mixture is the maintenance of the intestinal barrier, the production of antimicrobial peptides, and the reinforcement of the host immune system [3,6,40]. In all five studies [23,24,25,26,27] included, the administration of probiotic nutritional mixtures had an enhancing effect on anti-inflammatory cytokine profiles and discontinued any pro-inflammatory processes among patients with MS. In addition, the administration of such mixtures affected disability and mood levels as well as quality of life among patients with MS. More specifically, the scores observed in the BDI, DASS, and GHQ-28 scales were all significantly improved and thus provided promising data on the clinical impact of probiotic mixtures in several clinical aspects of MS via the modification of gut microflora. One study [23] provided data regarding the effect of nutritional probiotic mixtures on pain among patients with MS and concluded that the application of such mixtures may decrease the levels of pain as assessed using the Pain Rating Index (PRI). Such a result may provide encouraging data regarding pain management among patients with progressive MS [43]. Two research groups that attempted to provide direct disability data, as assessed using the EDSS scale, had mixed results: one study found a significant reduction in EDSS [24] among patients with MS and the other did not observe any significant effect [23]. Some of the applied probiotic nutritional mixtures may be found on the market as over the counter products, without being accompanied by any specific indication regarding MS treatment.

The concept behind FMT is to replace the whole gut microbiome with a healthy one, in order to restore its function. This therapeutic approach is quite effective in clinical conditions such as *Clostridium difficile* infection in inflammatory bowel disease (IBD) [44]. Regarding the efficacy of FMT in the clinical aspects of MS, further clinical investigation is necessary as the results were based on isolated patient cases. However, such protocols are highly interventional and have a significant financial burden; they may consequently not be easily adapted in day-to-day management among patients with MS.

Regarding calorie restriction with IF dietary regimes, the authors included two studies [30,31] which observed decreases in EDSS score as well as improvements in fatigue, sleep, and mood, with one achieving statistical significance [30]. Calorie restriction diet regimes with IF seem to exert a beneficial effect on autoimmune processes by reducing pro-inflammatory cytokines in EAE models [10]. Moreover, IF shifts the metabolic rate into a robust antioxidative profile via the modification of oxidative stress and mitochondrial function, thus contributing to improvements in physical and mental health [30,31]. Even though calorie restriction with IF seems quite demanding and may not be applicable among patients with MS with concomitant health issues such as glucose intolerance, it may be adopted quite easily as it does not require patients to have been suffering the condition for long; additionally, it is not financially burdensome.

## 5. Limitations

In this review paper, we attempted to investigate the possible impact of gut microflora modification through specific protocols under MS clinical parameters. Through this process, there were significant methodological limitations. The major methodological limitations included the absence of participants other than those diagnosed with RRMS and a very limited sample size in the majority of the studies. Other restricting factors included short study durations and the absence of objective clinical parameters for physical, mental, and cognitive assessment [19], as well as the underestimation of parameters such as cognitive impairment, which is a core feature of mainly progressive clinical forms of MS [24]. Finally, the majority of the data collected were derived from self-assessment patient questionnaires and not objective clinical evaluation scales [31].

## 6. Conclusions

The review article underlines the essential role of the gut microbiota on the clinical parameters of MS via the application of specific interventions with significant results in all studies presented as well as significant methodological limitations. Multitudes of commensal bacteria co-exist with hosts without incurring harmful immune responses. Symbiotic strains and their products are thus a precious mining pool that contains useful drug candidates with host-tolerated immune-modulatory functions. Innocuous commensal strains could also act as carriers for therapeutic substances when engineered. Finally, the use of fecal transplantation in restoring the richness and functionality of the gut microbial ecosystem has been proposed a long time ago; despite, methodological and ethical obstacles. Sufficiently funded, prospective longitudinal studies are needed to better understand the complex and likely dynamic relationship between MS, the gut microbiome, comorbidities, medication exposure, diet, and other lifestyle factors. 

## Figures and Tables

**Table 1:** Studies included in the review.

Author	Year	Region	Study Type	MS/Intervention	Control	Intervention Type
Borody	2011	USA	CR	3		FMT
Salami	2015	Iran	RCT	24	24	PNM
Yadav	2016	USA	RCT	32	29	DP
Saresella	2017	Italy	RCT	10	10	DP
Swidsinski	2017	Germany	CC	25	14	DP
Kouchaki	2017	Iran	RCT	30	30	PNM
Tankou	2018	USA	CC	9	13	PNM
Tankou	2018	USA	CC	9	13	PNM
Cignarella	2018	USA	RCT	8	8	IF
Fitzgerald	2018	USA	RCT	24	12	IF
Fard	2020	Iran	RCT	50	50	DP
Rahimlou	2022	Iran	RCT	35	35	PNM
Engen	2022	USA	CR	1		FMT

**Table 2:** Characteristics of included studies.

Dietary Protocols			
First Author	Sample-Duration	Results	Limitations
Fard et al. [19].	**100 RRMS** 50 participants on anti-inflammatory diet 50 participants in the control group—healthy diet 12 weeks	Significant improvements: **MFIS** scores (*p* = 0.001) **MSQoL-54: physical** component **(***p* = 0.015) **Mental** component (*p* = 0.003) in the diet group compared to the control group (*p* < *0.05*) significant increase in **IL-4** levels (*0.05*). **No significant changes** were observed in IL-17 and hs-CRP levels (*p* < *0.05*)	Short duration. Lack of more detailed scales to assess the effects of intervention on EDSS, psychological state, and cognitive function. Budget deficits in measurement of antioxidant vitamins.
Saresella et al. [20]	**20 RRMS** 10 participants on HV/LP diet 10 participants on Western diet 12 months	**rr** and **EDSS** reduced 12 m after HV/LP diet initiation	Baseline stool samples not collected.
Yadav et al. [21]	**61 RRMS** 32 participants on very-low-fat/plant-based diet 29 controls Patients randomized in very-low-fat/plant-based diet or placed on waitlist (control group) 1 year	Significant improvements in measures of **fatigue, BMI, and metabolic biomarkers** (*p = 0.001*)	Stringent inclusion criteria that allowed only people with RRMS and evidence of active disease in the previous 2 years. People interested in such a lifestyle intervention were mostly clinically stable patients or patients with progressive MS.
Swidsinski et al. [22]	**25 RRMS** 10 participants on Ketogenic diet 15 controls 6 months	In patients with MS, *Roseburia*, *Bacteroides*, *Faecalibacterium prausnitzii* probes were diminished the most. The effects of a ketogenic diet led to further reduction in bacterial concentrations and diversity.	Although the concentrations and the biodiversity of colonic microbiota are strong markers of the intensity of the microbial metabolism, the shifts in bacterial groups per se do not reveal the exact metabolic changes taking place.
**Probiotic Nutritional Mixtures**			
**First Author**	**n**	**Results**	**Limitations**
Rahimlou et al. [23]	**70 RRMS** 35 participants taking probiotic capsule (12 weeks) 35 participants taking placebo (6 months)	**BDNF** titer increase (0.001), **IL-6** titer decrease (0.001) **BDI** decrease (*p = 0.001*) **FSS** decrease (0.007)	Probiotic effects of HPA axis were not taken into account.
Salami et al. [27]	**48** patients with MS 24 participants taking probiotics 24 participants taking placebo 16 weeks	Significant differences in **EDSS**, **depression**, **anxiety**, and **stress** levels (*p =* <0.001) Also, significant differences in **CRP**, **IL-6, IL-10, NO** (*p* = <0.001)	Difficulty in counting microbial flora on the patients with MS.
Kouchaki et al. [24]	**60 RRMS** 30 participants taking probiotic capsule 30 participants taking placebo 12 weeks	Probiotic vs. placebo **EDSS** decrease (0.001) **DASS** decrease. (0.001) **GHQ** decrease (0.001) **MDI** decrease (0.001) Probiotic vs. placebo **hs-CRP** decrease (*p* = 0.01) **MDAI** decrease (*p* = 0.04) **NO** increase (*p* = 0.002) **Serum insulin, HOMA-IR, HOMA-B, HDL/TOTAL CHOL. Ratio** decrease (0.001, *p* = 0.001, *0*.001, *p* = 0.02) **QUICKI, HDL** increase (0.001)	No fecal bacteria load evaluation before and after probiotic supplementation. No assessment of other inflammatory markers. No evaluation of the effects of probiotic supplementation on cognitive impairment.
Tankou et al. [25]	**9 RRMS** (7 GA), **13 HCs** **LBS** probiotic mixture 2 months	LBS administration **taxa changes** in both MS and HCs microbiota (0.05) **KEGG** changes (0.05) **Stool metabolomics profile changes associated with decreased expression of MS risk allele** HLA.DPB1 (*p* = 0.078) HLA.DPA1 (*p* = 0.055) **Anti-inflammatory PBMC** immune response (*p* = 0.094)	Small sample size.
Tankou et al. [26]	**9 RRMS ** (7 GA), **13 HCs** **VSL3** probiotic mixture 2m	Increases in several species *(p = 0.048*); induction of anti-inflammatory IR (*p* = 0.08); VSL3 discontinued. Induction of pro-inflammatory IR (*p = 0.033*)	Small sample size.
**Fecal Microbiota Transplantation**			
**First Author**	**n**	**Results**	**Limitations**
Engen et al. [28]	**1 active RRMS**	**BDNF** titer increase (0.001) **12MSWS** score decrease. (0.05)	Single case report.
Borody et al. [29]	**3 “atypical” MS cases**	Case 1: **5** FMT improvement in mobility and urinary function Case 2: **10** FMT improvement in mobility and sensory function Case 3: **5** FMT improvement in bowel and mobility function	3 case reports.
**Intermittent Fasting**			
**First Author**	**n**	**Results**	**Limitations**
Cignarella et al. [30]	**16 RRMS (in relapse**) 8 IF 8 control diet 2 weeks	**EDSS** score decrease. No changes in **SDMT** and **MSFC** scores (no *p* values provided)	Pilot trial with short duration.
Fitzgerald et al. [31]	**36 RRMS** 12 Daily CR diet 12 IF CR diet 12 Weight-stable diet 8 weeks	**FAMS total** No change between groups *p* = 0.63 **FAMS—emotional wellbeing/depression** No change between groups *p* = 0.22 **FAMS—thinking/fatigue.** *p* = 0.53 **PSQI** No change between groups *p* = 0.38	Short duration. RRMS on injectable MS therapies. Patient self-report. Reduced adherence in IF compared with daily CR as a confounding factor.

BDI—Beck Depression Inventory; BDNF—brain-derived neurotrophic factor; BMI—body mass index; Chol—cholesterol; CRP—C-reactive protein; CR—calorie restriction; DASS—depression anxiety and stress scale; EDSS—expanded disability status scale; FAMS—functional assessment of multiple sclerosis; FSS—Fatigue Severity Scale; GA—glatiramer acetate; HC—healthy controls; HOMA B—homeostasis model of assessment (beta cell function); HOMA IR—homeostasis model of assessment (insulin resistance); HPA—hypothalamus–pituitary axis; HV/LP—high vegetable/low protein; IL—interleukin; IF—intermittent fasting; KEGG—Kyoto Encyclopedia of Genes and Genomes; LBS—lactobacillus–bifidobacterium–streptococcus; m—months; MDA—malondialdehyde; MDI—homeostasis model of assessment-estimated insulin resistance; MS—multiple sclerosis; 12MSWS— 12-item MS Walking Scale Assessment; MSFC—multiple sclerosis functional composite; MSQoL-54—Multiple Sclerosis Quality of Life; MFIS—Modified Fatigue Impact Scale, NO—nitric oxide; PBMC—frozen peripheral blood mononuclear cells; PSQI—Pittsburgh Quality Sleep Index; QoL—quality of life questionnaire; QUICKI—Quantitative Sensitivity Check Index; rr—annual relapse rate; RRMS—relapsing–remitting multiple sclerosis; SDMT—symbol digit modalities test; w—weeks.

## Data Availability

The datasets used and/or analyzed during the current study are available from the corresponding author on reasonable request.
